# Layer-Specific Vesicular Glutamate Transporter 1 Immunofluorescence Levels Delineate All Layers of the Human Hippocampus Including the *Stratum lucidum*

**DOI:** 10.3389/fncel.2021.789903

**Published:** 2021-12-09

**Authors:** Sarah Woelfle, Tobias M. Boeckers

**Affiliations:** ^1^Institute for Anatomy and Cell Biology, Ulm University, Ulm, Germany; ^2^International Graduate School in Molecular Medicine Ulm (IGradU), Ulm, Germany; ^3^Deutsches Zentrum für Neurodegenerative Erkrankungen (DZNE), Ulm, Germany

**Keywords:** human *post mortem* tissue, hippocampus, VGLUT1, VGLUT2, synaptoporin, immunofluorescence, infrapyramidal mossy fibers

## Abstract

The hippocampal formation consists of the Ammon’s horn (*cornu Ammonis* with its regions CA1-4), dentate gyrus, subiculum, and the entorhinal cortex. The rough extension of the regions CA1-3 is typically defined based on the density and size of the pyramidal neurons without clear-cut boundaries. Here, we propose the vesicular glutamate transporter 1 (VGLUT1) as a molecular marker for the CA3 region. This is based on its strong labeling of the *stratum lucidum* (SL) in fluorescently stained human hippocampus sections. VGLUT1 puncta of the intense SL band co-localize with synaptoporin (SPO), a protein enriched in mossy fibers (MFs). Owing to its specific intensity profile throughout all hippocampal layers, VGLUT1 could be implemented as a pendant to Nissl-staining in fluorescent approaches with the additional demarcation of the SL. Furthermore, by high-resolution confocal microscopy, we detected VGLUT2 in the human hippocampus, thus reconciling two previous studies. Finally, by VGLUT1/SPO co-staining, we provide evidence for the existence of infrapyramidal MFs in the human hippocampus and we show that SPO expression is not restricted to MF synapses as demonstrated for rodent tissue.

## Introduction

Excitatory signal conductance relies on the exocytotic release of glutamate from synaptic vesicles at the presynaptic terminal. Prior to transmitter release, vesicular glutamate transporters (VGLUTs) account for glutamate enrichment in synaptic vesicles. So far, three members of the VGLUT family have been identified, mostly termed VGLUT1, 2, and 3. VGLUT1 and VGLUT2 are broadly expressed in the brain with mostly complementary expression patterns. While VGLUT1 is enriched in the cerebral cortex, hippocampus, and cerebellum, VGLUT2 is mainly found in excitatory neurons of subcortical regions (Fremeau et al., 2001; Herzog et al., 2001). The detection of VGLUT3 at inhibitory synapses and in monoaminergic cells (Gras et al., 2002) suggested further implications of glutamate besides its role as transmitter of excitatory neurons. This holds also true for VGLUT1 and VGLUT2 that are not solely expressed in glutamatergic neurons (Zander et al., 2010; Heise et al., 2016). VGLUT1 and VGLUT2 have mainly been detected at neuronal terminals, while VGLUT3 has also been described in somata and at dendrites (Fremeau et al., 2002, 2004).

The brain-wide data sets unveiling the cellular and subcellular expression patterns were typically retrieved from rodent tissue upon the isolation of each transporter protein. Especially in the light of aberrant VGLUT1 and VGLUT2 expression in human neurological diseases like Alzheimer’s disease (AD) (Kirvell et al., 2006) and Parkinson’s disease (PD) (Kashani et al., 2007), a study from 2015 made a valuable contribution by providing VGLUT1-3 mRNA and protein expression data from a wide range of human *post mortem* brain regions, e.g., the hippocampus (Vigneault et al., 2015). Its essential role for learning and memory processes comes to light during its demise in the progression of AD.

While the DAB-stained overview sections serve as an ideal tool for the visual comparison of VGLUT1-3 protein expression, they do not allow for detailed, highly resolved inspection of the various hippocampal layers on synapse level. A study focusing on VGLUT1 expression in epileptic patients included high-resolution confocal images of CA3 and CA4 (van der Hel et al., 2009). In contrast to the study from 2015, they could not show detectable amounts of VGLUT2 in human control hippocampi. Therefore, given the known dominance of VGLUT1 in the hippocampal signal conductance (Herzog et al., 2006), the aim of this study was to expand the knowledge of VGLUT1 and VGLUT2 expression levels in the hippocampal layers by acquiring high-resolution confocal tilescans of human *post mortem* tissue. Besides, we found that another presynaptic protein, synaptoporin (SPO, also known as synaptophysin II), is expressed at VGLUT1-positive and -negative synapses.

## Materials and Methods

### Human Brain Samples

Human *post mortem* brain samples were obtained from permanent body donors of the gross anatomy course (Ulm University) as described elsewhere (submitted). Informed and written consent was obtained from all body donors. The study was approved by the ethics committee of Ulm University, Ulm.

Owing to the advanced age of the body donors, DAB-based staining for phospho-tau (clone AT8, 1:2000, Thermo Fisher Scientific, Cat. No. MN1020, RRID:AB_223647) and amyloid-β (clone 4G8, 1:5000, BioLegend, Cat. No. 800711, RRID:AB_2565324) was performed as previously described (Braak et al., 2011) to determine neuropathological AD stages (Thal et al., 2000; Braak et al., 2006). PD stages were determined in line with (Braak et al., 2003). For the present study, only low-staged cases were selected as presented in Supplementary Table 1.

From each case, a block from the hippocampal formation was excised at the level of the lateral geniculate nucleus. Afterwards, the excised blocks were cut with a vibratome (Microm HM 650 V, Thermo Scientific or VT1200S vibratome, Leica) to obtain 100 μm thick sections.

### Pigment Nissl Staining

100 μm thick, free-floating sections were stained with Darrow red (Sigma-Aldrich, Cat. No. 211885) and aldehyde fuchsine (Morphisto, Cat. No. 12763) to depict Nissl substance and lipofuscin, respectively (Braak, 1980; Braak et al., 1988). Afterwards, sections were dehydrated in an increasing alcohol series, refractive-index matched, and mounted as previously described (Feldengut et al., 2013).

Images were acquired in brightfield mode with a color camera of a Biorevo BZ-X810 microscope (Keyence). A 4× dry objective (CFl Plan Apo λ; numerical aperture (NA) 0.2; free working distance (WD) 20 mm) was used to capture the tiles.

### Immunofluorescence Staining

After cutting, the 100 μm thick hippocampal sections were rinsed three times with 1× Dulbecco’s phosphate-buffered saline (DPBS) (Gibco) plus 0.1% (vol/vol) Triton X-100 (Roche) (PBST) within one day. Prior to immunostaining on the next day, antigen retrieval was performed by boiling the tissue sections in 10 mM sodium citrate buffer (pH 8) for 20 min (water bath) as suggested previously for the detection of VGLUTs (Garcia-Marin et al., 2013). Thereafter, sections were cooled to room temperature and rinsed two times in 1 x DPBS. Sections were incubated in blocking solution (3% (wt/vol) bovine serum albumin (BSA) plus 0.3% (vol/vol) Triton X-100 in 1 × DPBS) for 3 h under gentle shaking at room temperature. Primary antibodies were diluted in blocking solution and applied for 48 h at 4°C under gentle shaking. The following primary antibodies were used: SPO-rabbit (1:200, Synaptic Systems, Cat. No. 102003, RRID:AB_2619748), VGLUT1-guinea pig (1:500, Synaptic Systems, Cat. No. 135304, RRID:AB_887878), VGLUT2-rabbit (1:200, Synaptic Systems, Cat. No. 135402, RRID:AB_2187539), and VGLUT2-mouse (1:100, Millipore, MAB5504, RRID:AB_2187552). Unbound antibodies were removed by rinsing the sections for 1 h and two times for 30 min with 1 x DPBS. Alexa Fluor (647, 488)-coupled secondary antibodies (Invitrogen and Jackson ImmunoResearch Laboratories, Inc.) were diluted 1:200 in blocking solution and incubated with the sections for 3 h at room temperature under gentle shaking. Afterwards, sections were rinsed again for 1 h and two times for 30 min. The DPBS solution for the first rinsing step included 4′,6-diamidino-2-phenylindole (DAPI, stock solution 10 mg/ml, diluted 1:10,000) for counterstaining of nuclei. Sections were mounted in ProLong Gold antifade reagent (Invitrogen), coverslipped, and cured overnight at room temperature. Negative controls with the omission of primary antibodies were included and yielded no signal (Supplementary Figure 4B).

Tilescans and high-resolution (2048 × 2048 pixels) z-stacks were acquired on a Leica SPE confocal microscope (Leica) with a 40× oil objective (ACS APO; NA 1.15; WD 270 μm). Excitation wavelengths of 635, 488, and 405 nm were used in the described order.

### Western Blot With Human Brain Lysate

Human frontal lobe lysate (Protein Medley, Takara, Cat. No. 635318) was diluted according to the manufacturer’s protocol. To avoid aggregation of VGLUTs, samples were not boiled. A protein ladder (PageRuler Prestained, Thermo Scientific) and the human sample (50 μg) were separated on a 4–15% precast gel (Bio-Rad, Cat. No. 4561085). Western blotting was done following standard protocols. The VGLUT2-rabbit antibody was applied at a concentration of 1:1000, β-actin (1:100,000, Sigma-Aldrich, Cat. No. A5316, RRID:AB_476743) served as loading control.

### Image Analysis

Confocal images were opened in Imaris (Oxford Instruments) software and adjusted for brightness and contrast. Finally, images were exported as TIFFs (stacks as maximum intensity projection) and figures were set up in Adobe Illustrator (Adobe). The overview scan acquired with the Biorevo microscope was stitched and brightness/contrast-adjusted in BZ-X800 Analyzer software (Keyence) and exported as TIFF. For intensity profiling, ImageJ (NIH) was used (plot profile for rectangle with width = 552 pixels). The raw values were exported to Excel (Microsoft Corporation), normalized to the maximum intensity, and graphs were generated in GraphPad Prism (GraphPad Software).

## Results

### Vesicular Glutamate Transporter 1 & 2 Antibody Selection and Inclusion Criteria for Human *post mortem* Tissue

Owing to the high conservation [human sequences: 76% amino acids identical (Almqvist et al., 2007)] between the three different VGLUT family members, antibodies were carefully selected based on manufacturer’s data and careful specificity testing. The applied VGLUT1 antibody is knock-out validated from manufacturer side and reactivity with the human protein is indicated. Moreover, immunostaining of the human basal ganglia showed the previously described (Vigneault et al., 2015) strong signal of VGLUT1 in the putamen and no expression in the pallidum (data not shown). Two different VGLUT2 antibodies were compared, one was raised in mouse, the other one in rabbit. According to the manufacturer’s website, the rabbit antibody is identical to the one used in a previous study showing no immunoreactivity in the human hippocampus (van der Hel et al., 2009). In general, reactivity with the human protein is indicated by the manufacturer and could be demonstrated in a western blot using human frontal lobe lysate (Supplementary Figure 4A). The mouse anti-VGLUT2 antibody was chosen because of its frequent application on human tissue and the herein assumed specificity.

Importantly, for the included body donors, no neurological disorder was documented and selection was based on the low AD and PD stage.

### Layer-Specific Pattern of Vesicular Glutamate Transporter 1 Protein Expression in the Human Hippocampus

To our knowledge, only few studies have depicted VGLUT protein expression in an entire human hippocampal section (e.g., van der Hel et al., 2009; Vigneault et al., 2015). In these studies, the method of choice for the overview images was DAB-based immunolabeling, suggesting high and layer-specific expression levels of VGLUT1. However, the extension of all layers did not become clearly recognizable in the low-resolved DAB overview sections.

**FIGURE 1:**
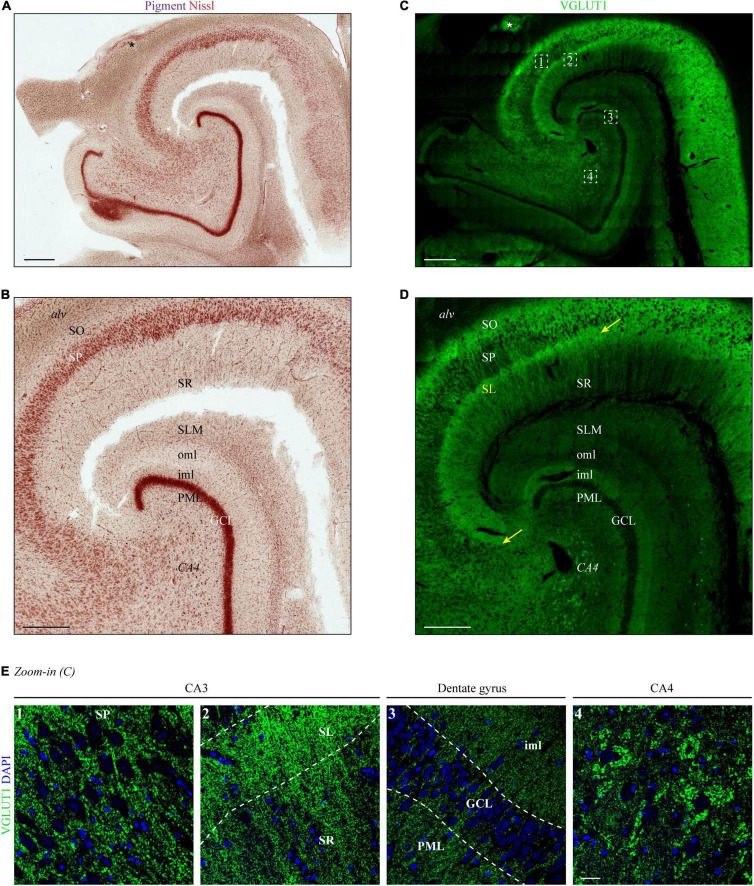
Layers of the human hippocampus determined by VGLUT1 immunofluorescence and Nissl staining. **(A)** Lipofuscin (pigment, purple) and Nissl substance (red) were stained with aldehyde fuchsine and Darrow red in a human hippocampal section, occipital part. The asterisk marks adjoining parts of the *gyrus fasciolaris*. Scale bar, 700 μm. **(B)** Zoom-in of panel **(A)** to display layer boundaries. *Alv* = *alveus*, SO = *stratum oriens*, SP = *stratum pyramidale*, SR = *stratum radiatum*, SLM = *stratum lacunosum-moleculare*, oml = outer molecular layer, iml = inner molecular layer, PML = polymorphic layer, GCL = granule cell layer, CA4 = *cornu Ammonis* region 4. Scale bar, 500 μm. **(C)** A subsequent section from the tissue block used in panel **(A)** was immunostained with VGLUT1 and acquired at a confocal microscope. LAS-X Navigator was used to create and stitch the tilescan. Dashed squares (white) are shown in panel **(E)** as high-resolution z-stacks. The asterisk marks adjoining parts of the *gyrus fasciolaris*. Scale bar, 700 μm. **(D)** Zoom-in of panel **(C)** to display layer boundaries. The yellow arrows mark the borders of the *stratum lucidum* (SL), i.e., CA3. Scale bar, 500 μm. **(E)** Maximum intensity projections (MIPs) of high-resolution z-stacks (stack size = 2.64 μm) acquired in the SP of CA3 (1), SL and SR of CA3 (2), dentate gyrus (3), and CA4 (4). Numbers refer to position 1–4 in the tilescan from panel **(C)**. Scale bar, 30 μm. Images in panels **(A–E)** are derived from case 1.

In a first step, we aimed to expand precision and resolution by performing immunofluorescence staining on entire human hippocampus sections with subsequent confocal microscopy. As a read-out for the determination of the hippocampal layers in VGLUT1-stained tissue sections, we performed traditional pigment Nissl staining on a subsequent human hippocampal section (Figure 1A). As shown by others (see, e.g., Braak and Del Tredici, 2015; Montero-Crespo et al., 2020), a higher magnified image (Figure 1B) allowed for a clear distinction of the following layers: *stratum oriens* (SO), *stratum pyramidale* (SP), *stratum radiatum* (SR), *stratum lacunosum-moleculare* (SLM), molecular layer of the DG, granule cell layer (GCL), and polymorphic layer (PML). Identification of the *stratum lucidum* (SL) and distinction between inner and outer molecular layer of the dentate gyrus (DG) (iml and oml, respectively) was not or hardly possible with the unaided eye.

Next, VGLUT1 immunofluorescence was acquired in a hippocampal section taken at the same level as the pigment Nissl-stained section. A tilescan (overview scan) encompassing CA1-4 and DG showed a clear expression gradient (Figure 1C), which permitted the allocation of layer boundaries in line with the Nissl staining.

In detail (see magnification of CA2-4 in Figure 1D), highest levels of VGLUT1 immunofluorescence were observed in the SP of CA2 and the adjoining SO (Figure 1D). An outstanding immunoreactive band could be detected at the supposed height of the SL underneath the SP (yellow arrows in Figures 1D,E, image two). This band became clearly apparent in all analyzed cases (yellow arrows in Figure 1D and Supplementary Figures 1A,B). Weaker, but still strong VGLUT1 expression was obtained in the SR, the iml, and the PML (Figure 1E, images two and three). Dim VGLUT1 fluorescence intensity was observed in the SLM and the oml (Figure 1D). Granule and pyramidal cell bodies were devoid of any VGLUT1 signal (Figure 1E, images one, three, and four), while perisomatic and peridendritic puncta were present especially at CA4 neurons (Figure 1E, image four). Owing to the specific fluorescence gradient along the layers (see Supplementary Figure 1D for intensity profiles), VGLUT1 staining permitted the additional identification of the SL/CA3 and the iml/oml border in the DG already with the unaided eye in contrast to conventional pigment Nissl staining.

### Confirmation of Layer Identity (*Stratum lucidum*) by Co-staining the Mossy-Fiber Enriched Protein Synaptoporin

Vesicular Glutamate Transporter 1 staining allowed for the identification of all hippocampal layers, including the SL. In contrast to the other layers, the extension of SL could not be validated with the corresponding pigment Nissl staining. Therefore, to confirm the assumed localization of SL in VGLUT1-stained sections, we co-stained tissue sections from two cases with an antibody against SPO, a protein enriched in mossy fibers (MFs), whose main termination site is the SL.

**FIGURE 2:**
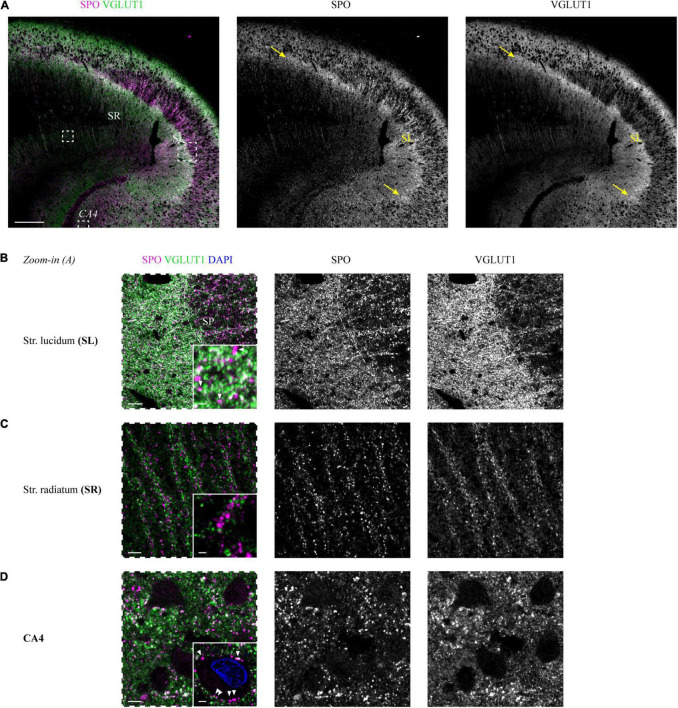
Co-staining of VGLUT1 and the mossy fiber-enriched protein synaptoporin in the human hippocampus. **(A)** Synaptoporin (SPO, magenta) and VGLUT1 (green) co-staining in a hippocampus section, areas of overlap appear in white (left). Individual channels are shown in the middle (SPO) and right (VGLUT1) column. Dashed squares (white) are shown in higher magnification in panels **(B–D)** as images with dashed frames. The yellow arrows mark the borders of the *stratum lucidum*, i.e., CA3. Scale bar, 300 μm. Zoom-in images of *stratum lucidum* **(B)**, *stratum radiatum* **(C)**, and CA4 **(D)** are displayed in the same manner as outlined in panel **(A)**. Contrast adjustment was performed identically for panels **(B–D)**. Insets at the lower right of each image represent MIPs of high-resolution z-stacks (z = 1.98 μm) in the respective layer, imaging parameters and contrast adjustments were kept constant. **(B)** White arrowheads in the inset mark non-co-localizing SPO puncta. Scale bar overview, 20 μm; inset, 2 μm. **(C)** Scale bar overview, 10 μm; inset, 2 μm. **(D)** White arrowheads in the inset mark perisomatic SPO puncta of a mossy cell in CA4. Scale bar overview, 10 μm; inset, 3 μm. Images in panels **(A–D)** are derived from case 1.

The immunoreactive band that became apparent in the VGLUT1 channel underneath the SP was highlighted in an identical extension with the SPO antibody (white overlap in Figure 2A). This finding could be replicated for the second tested case (Supplementary Figure 2A).

### Expression of Synaptoporin Beyond Vesicular Glutamate Transporter 1-Positive Mossy Fiber Synapses in the Human Hippocampus

Exploiting the VGLUT1 overview scans shown in Figure 2A and Supplementary Figure 2A, we analyzed SPO co-expression in higher resolved acquisitions from all hippocampal layers. So far, SPO expression has mainly been analyzed in selected human hippocampal layers, mostly in the context of epilepsy (see, e.g., Janz et al., 2017; Schmeiser et al., 2017; Freiman et al., 2021).

Starting with the SL, the high overlap seen in the overview scan could be confirmed. However, in the higher resolved images, SPO-positive puncta without VGLUT1 co-localization were present (white arrows in insets of Figure 2B and Supplementary Figure 2B). In the SR, SPO puncta were less dense and did not co-localize with VGLUT1 puncta (Figure 2C and Supplementary Figure 2C). SPO puncta were found around the somata (arrows in the inset of Figure 2D) and apical dendrites of CA4 mossy cells (arrows in the inset of Supplementary Figure 2D).

**FIGURE 3:**
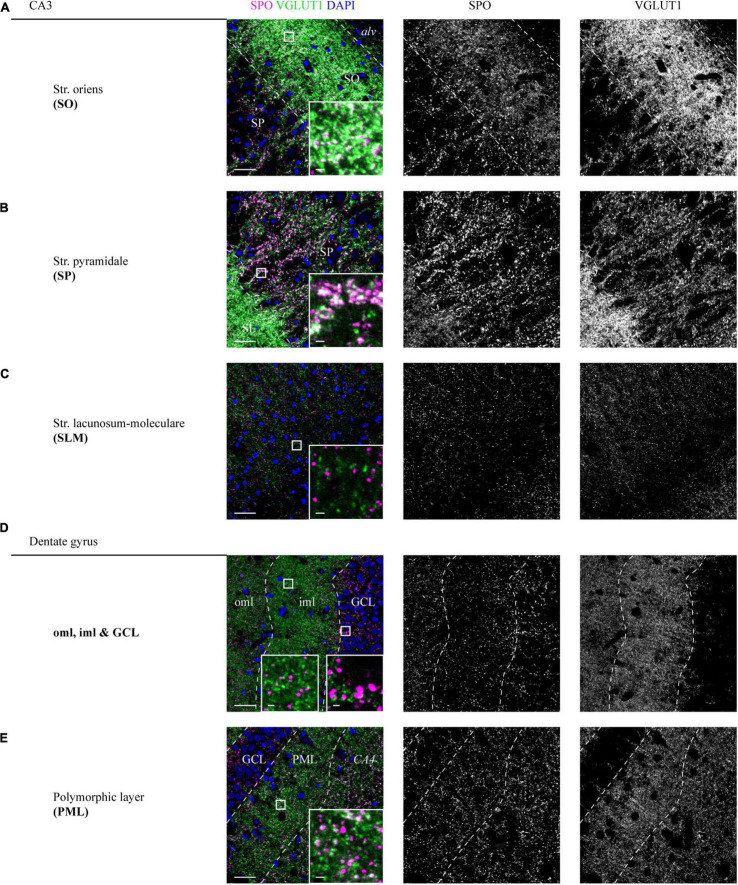
Distribution of SPO at VGLUT1-positive synapses in CA3 and DG layers of a human hippocampus. **(A–E)** High-resolution z-stacks (z = 1.98 μm) were acquired in a hippocampus section co-stained for SPO and VGLUT1. A tilescan of the VGLUT1 channel served as a map to identify the layers and define the z-stacks. Overlay of SPO (magenta), VGLUT1 (green), and DAPI (blue) is shown in the left, individual channels are shown in the middle (SPO) and right (VGLUT1) column. White squares are shown as higher magnified insets at the lower right of each image. Imaging parameters and contrast adjustments were kept constant for all images. Scale bars overviews, 30 μm; scale bars insets, 2 μm. Images in panels **(A–E)** were derived from case 1.

In the SO, a high overlap between SPO and VGLUT1 puncta could be detected (insets, Figure 3A and Supplementary Figure 3A). In line with the expression in the SL, VGLUT1 and SPO mainly co-localized in the adjoining SP (insets, Figure 3B and Supplementary Figure 3B). In the SLM, SPO expression was weak and no obvious co-localization with VGLUT1 could be observed (insets, Figure 3C and Supplementary Figure 3C). All layers of the CA were analyzed at the level of CA3.

In the DG, SPO was expressed in all layers without a characteristic pattern as seen for VGLUT1 (Figures 3D,E and Supplementary Figures 3D,E). No co-localization of SPO was observed in the oml, iml, and GCL (insets, Figure 3D and Supplementary Figure 3D), whereas in the PML, a fraction of spots clearly co-localized (insets, Figure 3E and Supplementary Figure 3E).

### Detectable Vesicular Glutamate Transporter 2 Immunoreactivity in the Human Hippocampus

Besides VGLUT1, we aimed to perform VGLUT2 immunostaining in the three cases used for VGLUT1 staining to reconcile previous reports about expression patterns in the human hippocampus. First, we applied the VGLUT2-rabbit antibody. In contrast to VGLUT1 overview scans, VGLUT2 did not permit clear distinction of all layers (Supplementary Figure 1C). Co-staining with VGLUT1 in a second channel was thus again helpful for the navigation through the different layers: In the low-resolving tilescan, high VGLUT2 signals were obtained in the somatic layers (SP and GCL). Besides, enhanced immunoreactivity was found in the SLM and the molecular layer of the DG, comparable to rodent overview scans (see, e.g., Heise et al., 2016). Expression levels of VGLUT1 and VGLUT2 in the SLM are in good agreement with the overall reported complementary expression.

**FIGURE 4:**
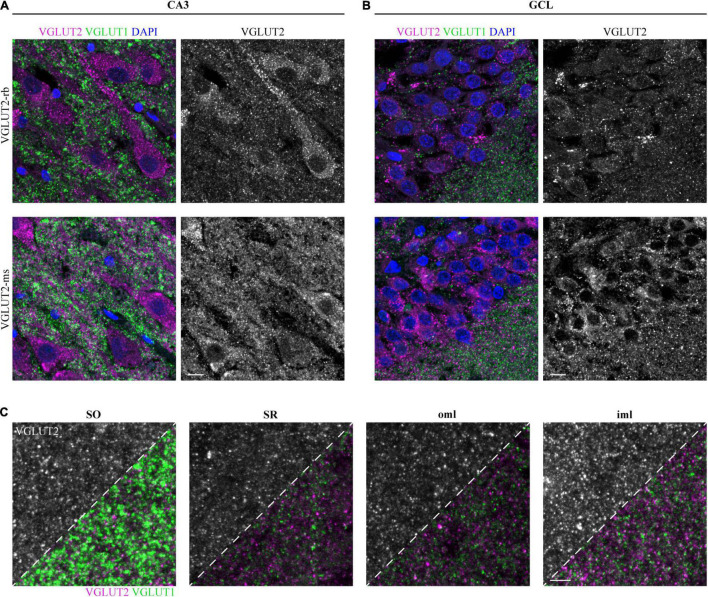
Detectable VGLUT2 immunofluorescence in the human hippocampus. **(A)** Immunofluorescence staining with VGLUT2 (magenta) and VGLUT1 (green) in the SP of CA3 is represented as overlay (left) and separately for VGLUT2 (gray, right). Staining results obtained with two different VGLUT2 antibodies are opposed, one antibody was raised in rabbit (upper row), the second one was raised in mouse (lower row). Images represent MIPs of high-resolution z-stacks (z = 1.98 μm), imaging parameters and contrast adjustments were kept constant per antibody. **(B)** In the same manner as explained in panel **(A)**, immuno-reactivity for the GCL is displayed. Scale bars **(A,B)**, 10 μm. **(C)** Rabbit anti-VGLUT2 immunofluorescence in the SO, SR, oml, and iml. Acquired images are shown in a dual fashion; the upper left corner solely shows the VGLUT2 channel, for the lower right corner, the VGLUT1 channel was overlaid. Images represent MIPs of z-stacks (z = 1.98 μm) and imaging parameters as well as contrast adjustments are constant per antibody. Co-staining did not reveal co-localizing puncta, arguing for specific labeling of both proteins. Scale bar, 5 μm. Images in panels **(A,B)** are derived from case 1, **(C)** represents case 2.

In contrast to VGLUT1, somatic puncta were detected in the pyramidal neurons of CA3 (Figure 4A, upper row) and the granule cells of the GCL (Figure 4B, upper row). Of note, VGLUT2 puncta were closely aligned along the apical dendrites (Figure 4A, upper row). To confirm these results, subsequent hippocampal sections were fluorescently labeled with a second VGLUT2 antibody, which was raised in mouse and applied to human tissue in the past (e.g., Garcia-Marin et al., 2013). Similar to the rabbit anti-VGLUT2 antibody, somatic and peridendritic staining in CA3 (Figure 4A, lower row) and somatic labeling in the GCL (Figure 4B, lower row) was observed. This finding was persistent also with higher antibody dilutions (Supplementary Figures 4C,D). Puncta in the neuropil were found in several layers. In our hands and with the applied protocol, the rabbit antibody performed better with respect to background and penetration depth. We therefore used the sections stained with this antibody to acquire layer-specific images and to compare intensities.

Apart from the somatic layers, which were clearly highlighted in the tilescan, we could detect VGLUT2 expression in additional layers, e.g., SO, SR, oml, and iml (Figure 4C). Among the displayed layers, VGLUT2 puncta were most intense in the iml (Figure 4C, left half of each image).

## Discussion

In this study, we showed that VGLUT1 immunofluorescence can serve as a molecular marker for the CA3 region of the human *cornu Ammonis* through its enrichment at glutamatergic terminals (MFs) in the *stratum lucidum*. In contrast to the MF-enriched protein SPO or non-immuno-based detection techniques (e.g., via fiber-enriched zinc in Timm’s staining), VGLUT1 additionally allowed for the demarcation of all hippocampal layers of a CA region and the DG. Thus, VGLUT1 immunofluorescence unites classical Nissl staining (identification of most layers) and vesicle-specific immunostaining (MFs, SL) to identify all hippocampal layers. Importantly, layer identification was already feasible without quantitative analysis. Second, we observed detectable levels of VGLUT2 in the human hippocampus, which was not clearly shown in previous studies. Finally, by co-staining VGLUT1 and SPO, we visualized infra- and suprapyramidal MFs and observed SPO puncta in hippocampal layers beyond the MF system.

The human *hippocampus proper* consists of four regions: while CA4 can be easily identified in the hilus, CA1-CA3 are merely histologically characterized based on the size and density of the pyramidal neurons. Thus, the definition of clear-cut borders is hardly possible. The so-called trisynaptic circuit describes an essential flow of information, starting with excitatory inputs from projection neurons of the entorhinal region (perforant path) to the outer two thirds of the DG molecular layer (oml). The second connection is established by so-called MFs of DG granule cells, whose enormous boutons terminate on proximal dendrites of pyramidal neurons in CA3 [or, more precisely, its thorny excrescences (Amaral and Dent, 1981)]. Unique for CA3, the termination site of MFs at the apical dendrites is known as SL. In turn, axons from CA3, called Schaffer collaterals, target the SO and SR of CA1, thereby constituting the third synapse of the circuit (Amaral and Witter, 1989; Braak et al., 1996).

The presynaptic transporter protein VGLUT1 is a popular marker for labeling glutamatergic synapses. VGLUT1 protein expression has already been analyzed in human hippocampi by acquiring overview scans of DAB-stained tissue sections (van der Hel et al., 2009; Vigneault et al., 2015). Here, by performing high-resolution confocal microscopy of fluorescently-labeled hippocampus sections, a striking immunoreactive band underneath the supposed position of CA3 was highlighted by the applied VGLUT1 antibody, which appeared more pronounced than seen in comparable DAB sections. Its punctate pattern most likely represents the excitatory terminals from granule cells of the DG, which form the SL. In support, previous studies in rat have shown VGLUT1 expression in the SL (Fremeau et al., 2001; Williams Megan et al., 2011). The gradient of VGLUT1 fluorescence along the other hippocampal layers described here, was in line with previous studies on human tissue (e.g., van der Hel et al., 2009). Of note, the pigment Nissl-stained section did not allow for the distinction of the iml and oml with the unaided eye and, most important, did not delineate the SL. Co-staining of SPO (also known as synaptophysin II) confirmed the identity of the SL.

When describing the SL as termination site of MFs, we actually refer to the subset of suprapyramidal MFs. Besides, so-called infrapyramidal MFs were found in the SO (West, 1983; Römer et al., 2011). This concept is most likely reflected in the present study by the presence of SPO/VGLUT1-positive puncta not only in the SL, but also in the SO of CA3. MFs along the whole dendrite extending in the SP of CA3 were described as “intrapyramidal MFs” (Singec et al., 2002) in rodent tissue and could be also observed in the present study, mostly co-localizing with VGLUT1.

Apart from CA3, MFs terminate in CA4 and the PML of the DG (Braak et al., 1996). In agreement with the primarily excitatory nature of MFs, SPO puncta were found to be positive for the excitatory marker VGLUT1 in the aforementioned three areas. In the SR, SLM, and molecular layer of the DG, SPO seems to be mostly expressed at inhibitory synapses, which was shown by a clear segregation of VGLUT1 and SPO puncta in these layers [same results were reported for SR and SLM in rodent tissue (Williams Megan et al., 2011)]. On the contrary, even though the bulk (∼80%) of glutamate transport is mediated by VGLUT1 (Fremeau et al., 2004; Vigneault et al., 2015), it might be conceivable that SPO-positive/VGLUT1-negative puncta, e.g., in the molecular layer of the DG, are not solely positive for primarily inhibitory markers (for rodent tissue, co-localization of GAD65 and SPO was shown in oml, e.g., see Singec et al., 2002), but partly co-localize with other VGLUT family members [e.g., VGLUT2 was found to be enriched in the molecular layer of the DG (Vigneault et al., 2015)].

SPO expression levels in all layers of the mouse and rat hippocampus have been previously compared (Singec et al., 2002). While the overview scans from rodent tissue show layer-specific differences in the molecular layer of the DG (mouse: iml stronger than oml, rat: iml ∼ oml) or in SR and SLM of CA1 (mouse: SR stronger, rat: SLM stronger), we did not see obvious expression gradients in the DG, but the SR appeared more pronounced than the SLM in our human samples (CA1, data not shown). Inhibitory terminals, which were found to partially co-localize with SPO in rodent tissue (Singec et al., 2002), terminate at the axon, soma, or dendrite of their target cell, depending on the transmitter. Here, a fraction of SPO puncta in CA4, which were smaller than the striking MF boutons, showed a perisomatic and -dendritic localization, putatively representing VGLUT1-negative inhibitory synapses.

Taken together, we provide a detailed map for SPO in all layers of the human *hippocampus proper* (CA3 region) and DG and we could show by VGLUT1 co-staining that SPO is present at both, excitatory and VGLUT1-negative synapses. Expression at VGLUT1-positive synapses was confined to layers receiving input from MFs.

While our VGLUT1 pattern is well comparable to previous studies, data on VGLUT2 obtained with different antibodies are contradictory. In the more recent human study, a homemade antibody detected VGLUT2 in the molecular layer of the DG, the MF pathway, and the subiculum (Vigneault et al., 2015). In previous studies using a commercially available antibody, VGLUT2 could not be detected in the human hippocampus by DAB-based staining (van der Hel et al., 2009), most likely due to technical reasons. With the aim to reconcile both studies, we applied a commercial antibody at a lower dilution and with the previously suggested (Garcia-Marin et al., 2013) antigen retrieval prior to immunolabeling. Indeed, we could show VGLUT2 puncta in the neuropil of several hippocampal layers as well as in the soma of pyramidal and granule cells. A second VGLUT2 antibody previously applied in human tissue lead to the same finding. A closer look into some of the rodent studies unveils discrepancies as well: Several studies reported VGLUT2 protein expression concordantly in the GCL of the DG (Fremeau et al., 2001; Herzog et al., 2006; van der Hel et al., 2009), but its detection in the molecular layer of the DG (Herzog et al., 2006; van der Hel et al., 2009) and the SLM of CA1 (Fremeau et al., 2001; Herzog et al., 2006) is not consistently mentioned. A more recent study on mouse tissue (Heise et al., 2016) has included detailed extracts of most hippocampal layers, which were acquired with a confocal microscope. VGLUT2 was most pronounced in the somatic layers, but the images clearly demonstrated the presence of VGLUT2 puncta throughout many layers, albeit mostly in low quantities. Thus, the putatively weak expression of VGLUT2 in most hippocampal layers might have contributed to some discrepancies in past studies.

Besides the punctate staining pattern in the neuropil, both VGLUT2 antibodies showed somatic puncta in the pyramidal cells of CA3 and the granule cells of the DG. This finding was derived from two different antibodies and could be confirmed in single stainings with a series of antibody dilutions. While VGLUT2 signals from the GCL have not always been assigned to the somata (e.g., Fremeau et al., 2001), studies conducted on mouse tissue reported intracellular protein labeling in combination with *in situ* hybridization (Agis-Balboa et al., 2006, 2007). Hence, additional studies should elucidate whether these findings can be confirmed with tissue from younger donors, if possible. Due to the high cell density in the GCL, co-staining with a cellular antibody might be of help in future studies to distinguish the VGLUT2 signal at terminals from intracellular, VGLUT2-positive puncta. Moreover, permeabilization of the sections and antigen retrieval might be required to facilitate VGLUT2 detection. One group, who used the same VGLUT2-mouse antibody as applied here reported inconsistent results without applying heat-mediated antigen retrieval prior to VGLUT2 immunohistochemistry on macaque and human sections (Garcia-Marin et al., 2013). Finally, VGLUT2 protein expression presumes the existence of *VGLUT2* mRNA in pyramidal cells and the DG. Existing data from human, mouse, and rat tissue suggest minor levels of *VGLUT2* mRNA [e.g., (Ziegler et al., 2002; McCullumsmith and Meador-Woodruff, 2003) or The Human Protein Atlas^1^ ]. Owing to the conflicting nature of the current literature, future experiments should ideally address mRNA expression on single cell level in human *post mortem* tissue to precisely allocate previously detected mRNA, which, however, might be a challenging endeavor in paraformaldehyde-fixed tissue.

Besides somatic labeling, both VGLUT2 antibodies unveiled peridendritic and astrocytic labeling (data not shown). Since GABAergic contacts in the SP are preferentially formed at cell somata and proximal dendrites, peridendritic VGLUT2 might represent inhibitory terminals in line with a previous report (Zander et al., 2010). So far, conflicting data exist about the astrocytic localization of VGLUTs *in situ*, e.g., while initial reviews mainly assigned VGLUT3 to astrocytes (Fremeau et al., 2004), additional work reported VGLUT1 and VGLUT2 expression in astrocytes of the hippocampus (Bezzi et al., 2004). On the contrary, a more recent and extensive study concluded that the protein family is absent in astrocytes (Li et al., 2013). Of note, it was not explicitly reported on astrocyte/VGLUT2 co-staining in the mouse hippocampus. Thorough analysis of mRNA expression would clarify the issue whether potential VGLUT2 protein in scattered astrocytes is found because of cell-autonomous expression or as a result of phagocytosed extrinsic material (Lee et al., 2021).

In conclusion, future studies in hippocampal tissue should exploit VGLUT1 as a molecular marker for the CA3 region of the *cornu Ammonis* and implement VGLUT1 co-staining as Nissl staining for fluorescent approaches. SPO/VGLUT1 co-staining showed the existence of a diversely organized pre-synaptic machinery in the human hippocampus. Given the strong signal of VGLUT1 already at low antibody concentrations, even autofluorescence-prone short wavelengths (e.g., excitation at 488 nm) can be exploited for its detection.

## Data Availability Statement

The original contributions presented in the study are included in the article/Supplementary Material, further inquiries can be directed to the corresponding authors.

## Ethics Statement

The studies involving human participants were reviewed and approved by Ethics Committee of Ulm University, Ulm. The patients/participants provided their written informed consent to participate in this study.

## Author Contributions

SW: study concept, experiments, data analysis, and first draft of manuscript. TB: manuscript. Both authors contributed to the article and approved the submitted version.

## Conflict of Interest

The authors declare that the research was conducted in the absence of any commercial or financial relationships that could be construed as a potential conflict of interest.

## Publisher’s Note

All claims expressed in this article are solely those of the authors and do not necessarily represent those of their affiliated organizations, or those of the publisher, the editors and the reviewers. Any product that may be evaluated in this article, or claim that may be made by its manufacturer, is not guaranteed or endorsed by the publisher.

## Footnotes

1
https://www.proteinatlas.org/ENSG00000091664-SLC17A6/brain

## Abbreviations

AD: Alzheimer’s disease
CA: *cornu Ammonis*
DG: dentate gyrus
GCL: granule cell layer
iml: inner molecular layer of the DG
MF: mossy fiber
oml: outer molecular layer of the DG
PD: Parkinson’s disease
PML: polymorphic layer
SL: *stratum lucidum*
SLM: *stratum lacunosum-moleculare*
SO: *stratum oriens*
SP: *stratum pyramidale*
SPO: synaptoporin
SR: *stratum radiatum*
VGLUT: vesicular glutamate transporter.
