# Supplementary material for: Layer-Specific Vesicular Glutamate Transporter 1 Immunofluorescence Levels Delineate All Layers of the Human Hippocampus Including the Stratum lucidum
Source: Front Cell Neurosci. 2021 Dec 9;15:789903. doi: 10.3389/fncel.2021.789903 (PMC8696355; doi:10.3389/fncel.2021.789903)
Supplement: Supplementary file 1 [file Data_Sheet_1.docx]

Supplementary Material

# Supplementary Figures and Tables

## Supplementary Figures


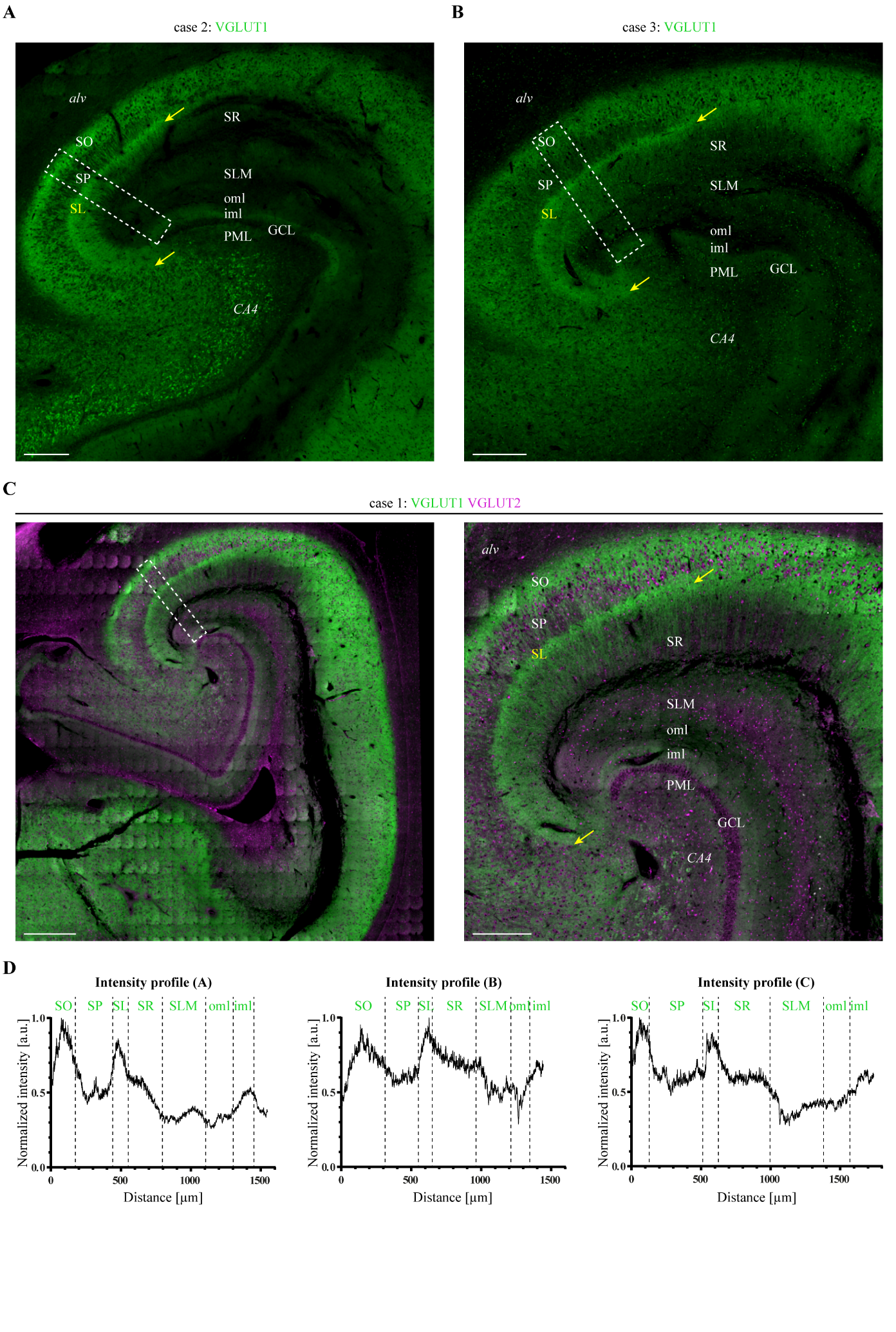


**Supplementary Figure 1.** **Demarcation of hippocampal layers by VGLUT1 immunofluorescence and VGLUT2 co-staining. (A)** Tilescan of VGLUT1 immunofluorescence in a hippocampal section from case 2; scale bar, 500 µm and **(B)** from case 3; scale bar, 500 µm. The yellow arrows mark the borders of the *stratum lucidum* (SL), i.e. CA3. **(C)** VGLUT1/VGLUT2 co-staining (case 1), shown as overview scan (left) and in higher magnification (right). The yellow arrows mark the borders of the *stratum lucidum* (SL), i.e. CA3. Scale bars, 1 mm (left), 500 µm (right). **(D)** Normalized VGLUT1 intensity profiles of the boxed areas in (A)-(C). Plots were generated on the raw images, which had been rotated before placing a horizontal rectangular selection in ImageJ. Peaks were found in the SO and the SL. Comparably high, but lower levels were detected in the SR and the iml. A.u. = arbitrary unit.


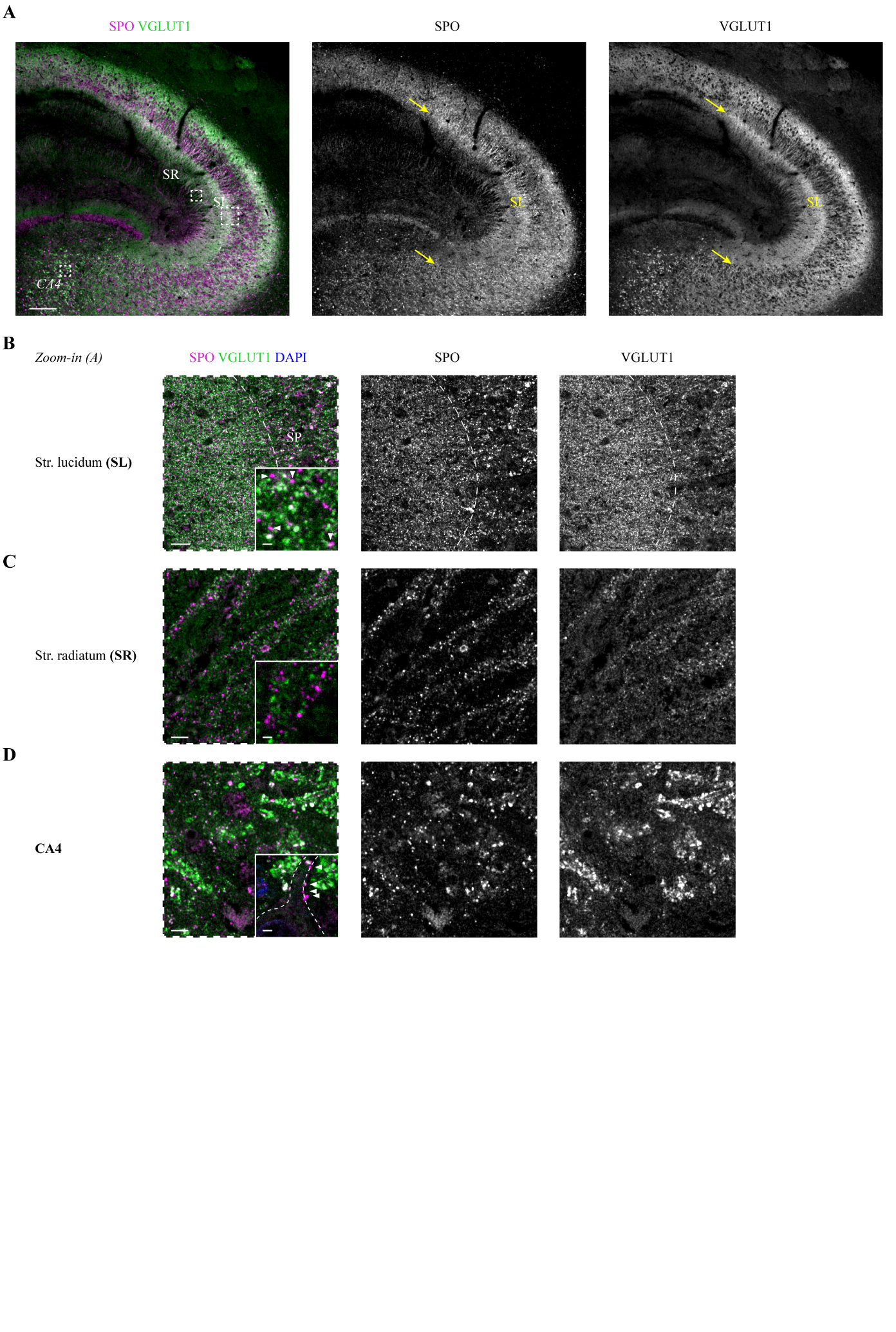


**Supplementary Figure 2. Co-staining of VGLUT1 and SPO in a human hippocampus section from case 2. (A)** SPO (magenta) and VGLUT1 (green) co-staining in a hippocampus section, areas of overlap appear in white (left). Individual channels are shown in the middle (SPO) and right (VGLUT1) column. Dashed squares (white) are shown in higher magnification in (B)-(D) as images with dashed frames. The yellow arrows mark the borders of the *stratum lucidum*, i.e. CA3. Scale bar, 300 µm. Zoom-in images of *stratum lucidum* **(B)**, *stratum radiatum* **(C)**, and CA4 **(D)** are displayed in the same manner as outlined in (A). Contrast adjustment was performed identically for (B)-(D). Insets at the lower right of each image represent MIPs of high-resolution z-stacks (z = 1.98 µm) in the respective layer, imaging parameters and contrast adjustments were kept constant. **(B)** White arrowheads in the inset mark non-co-localizing SPO puncta. Scale bar overview, 20 µm; inset, 2 µm. **(C)** Scale bar overview, 10 µm; inset, 2 µm. **(D)** White arrowheads in the inset mark pericellular SPO puncta along an apical dendrite of a mossy cell in CA4. Scale bar overview, 10 µm; inset, 3 µm.


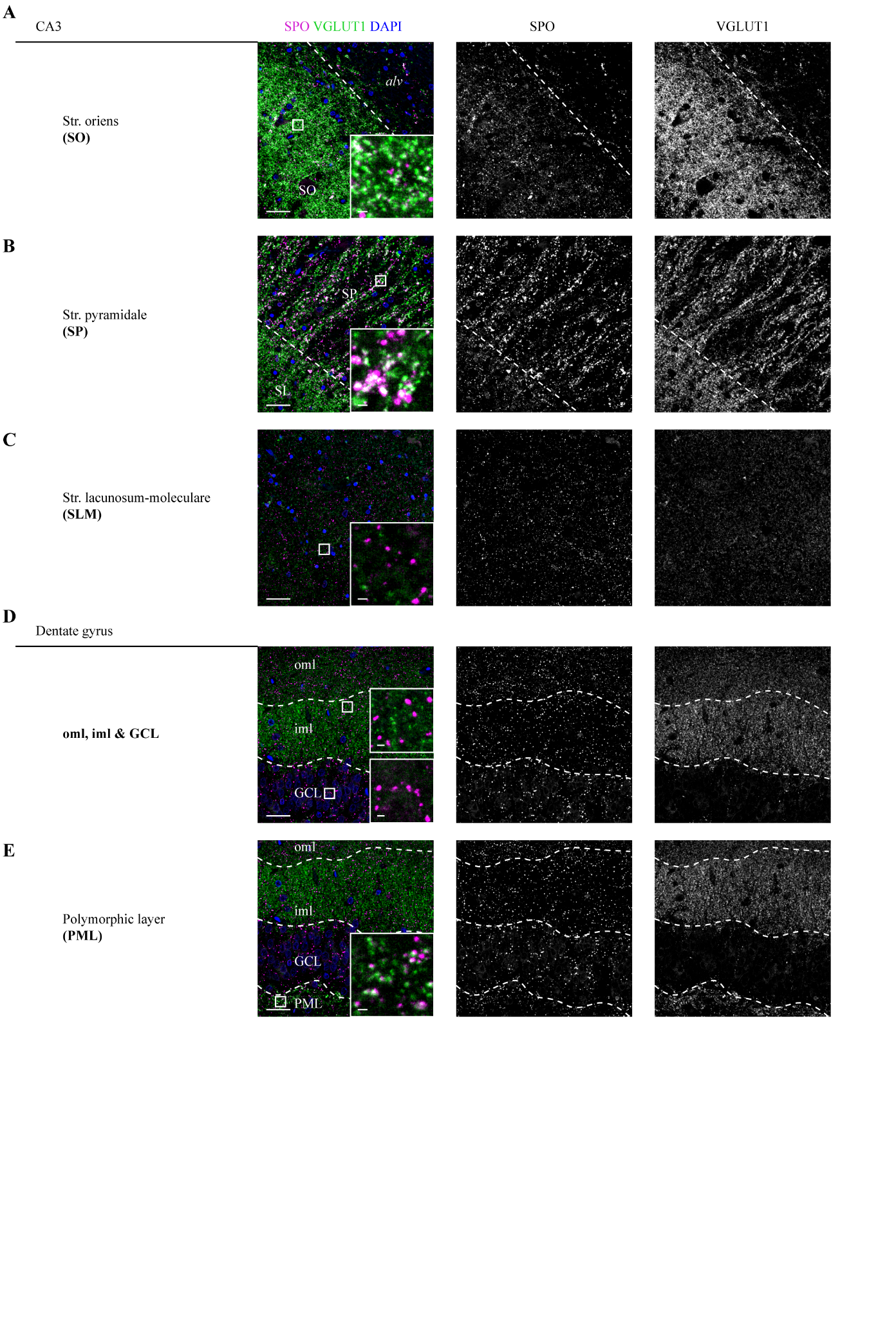


**Supplementary Figure 3. Distribution of SPO at VGLUT1-positive synapses in CA3 and DG layers of a human hippocampus, case 2. (A)-(E)** High-resolution z-stacks (z = 1.98 µm) were acquired in a hippocampus section co-stained for SPO and VGLUT1. A tilescan of the VGLUT1 channel served as a map to identify the layers and define the z-stacks. Overlay of SPO (magenta), VGLUT1 (green), and DAPI (blue) is shown in the left, individual channels are shown in the middle (SPO) and right (VGLUT1) column. White squares are shown as higher magnified insets at the lower right of each image. Imaging parameters and contrast adjustments were kept constant for all images. Scale bar overviews, 30 µm; scale bars insets, 2 µm.


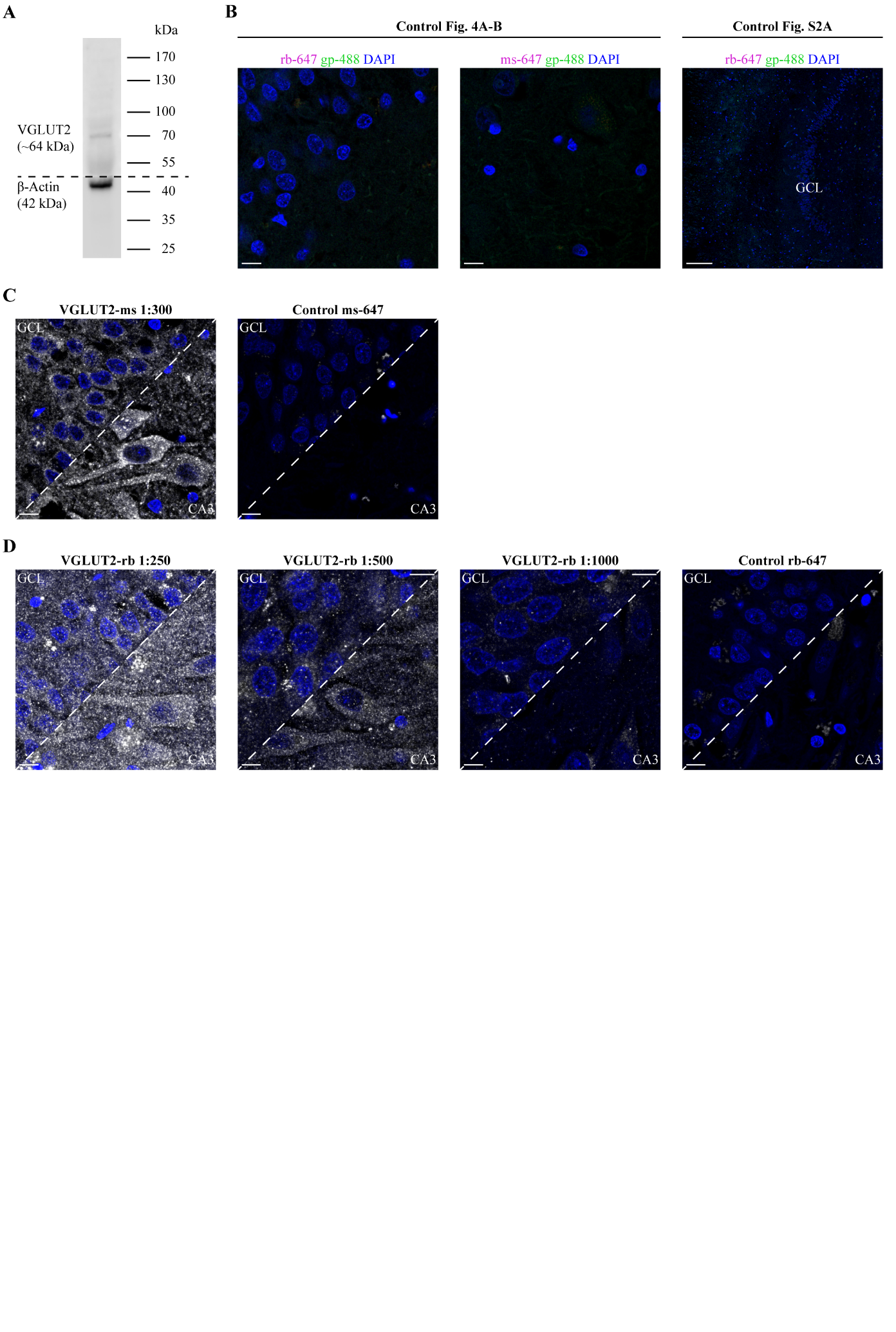


**Supplementary Figure 4. Persistent somatic and peridendritic staining in a series of VGLUT2 antibody dilutions.** **(A)** Western blot with the VGLUT2-rabbit antibody showed a specific signal at the expected height in human frontal lobe lysate. kDa = kilodalton. **(B)** Exemplary negative controls for Figures 4 and S2. Secondary-only controls were acquired and processed identically as the actual staining. Scale bars left and middle, 10 µm; right, 150 µm. **(C)** Highest recommended dilution for the VGLUT2-ms antibody showed persistent somatic and peridendritic staining. **(D)** Dilution series for the VGLUT2-rb antibody. All dilutions were acquired and processed with identical parameters. Images in (C) and (D) represent MIPs of high-resolution z-stacks (z = 1.98 µm). The secondary-only controls were acquired and processed identically as the actual staining. Scale bars, 10 µm, (D) 1:500 & 1:1000 GCL, 8 µm. Images in (C)-(D) are derived from case 2. Rb = rabbit, gp = guinea pig, ms = mouse.

## Supplementary Table

Table 1 Details about study cases

| **Case** | **Sex** | **Age** | **Cause of death** | **AD stage (NFT/Aβ)** | **PD stage** |
| --- | --- | --- | --- | --- | --- |
| 1 | male | **71** | Cardiogenic shock | I/0 | 0 |
| 2 | male | **74** | Decompensated heart insufficiency | I/3 | 0 |
| 3 | male | **89** | Myocardial infarct | I/3 | 1 |

AD = Alzheimer’s disease, PD = Parkinson’s disease, NFT = neurofibrillary tangle, Aβ = amyloid-β
